# Effects of Nano-Iron Sulfide on Growth Performance, Antioxidant Status, Meat Quality, and Intestinal Microbiota in Broilers, and Tolerance Evaluation

**DOI:** 10.3390/ani16172660

**Published:** 2026-08-25

**Authors:** Tongtong Wang, Jiahao Tang, Qianxi Li, Lizeng Gao, Bing Dong

**Affiliations:** 1 State Key Laboratory of Animal Nutrition and Feeding, College of Animal Science and Technology, China Agricultural University, Beijing 100083, China; ttwangsd23@163.com (T.W.); tjh2057@163.com (J.T.); liqianxi2022@163.com (Q.L.); 2 CAS Engineering Laboratory for Nanozyme, State Key Laboratory of Biomacromolecules, Institute of Biophysics, Chinese Academy of Sciences, Beijing 100083, China; gaolizeng@ibp.ac.cn

**Keywords:** nano-iron sulfide, bioavailability, growth performance, antioxidant, microbiota, tolerance

## Abstract

This study evaluated nano-iron sulfide (nFeS) as an iron source for bioavailability, biocompatibility, and tolerance in broilers. Broilers (Arbor Acres; males; initial body weight: 43.0 ± 0.4 g) were allocated between five diets: an iron-deficient basal diet (ID), or a basal diet with 80 mg Fe/kg as FeSO_4_; or 20, 40, or 80 mg Fe/kg as nFeS. The results showed that nFeS treatments (40, 80 mg/kg) improved average daily gain and feed conversion rates compared to ID and showed comparable or even superior effects relative to FeSO_4_ on growth performance within 1–21 days (*p* < 0.05). nFeS improved whole-blood hemoglobin (Hb) content and packed cell volume (PCV) percentages at day 21, while increasing serum SOD and GSH-Px activities at day 42. nFeS (40, 80 mg/kg) had or tended to deposit higher iron content in the liver, spleen, duodenum and serum than the FeSO_4_ groups. nFeS significantly increased breast meat lightness and redness compared with ID (*p* < 0.05) and showed comparable effects on postmortem pH, drip loss, and shear force relative to FeSO_4_. nFeS reshaped the microbial composition in the jejunum and cecum, particularly enriching the abundance of beneficial bacteria such as *Lactobacillus* in the jejunum. At a dose of up to 400 mg/kg, nFeS revealed no negative effects in broilers. In conclusion, nFeS is an effective iron supplement with high bioavailability and comparability, with additional benefits in shaping intestinal microbiota.

## Simple Summary

Iron is an essential mineral for broiler growth due to its oxygen-carrying function and maintenance of energy homeostasis. Plant-based diets supplemented with inorganic iron are commonly applied in broiler feed because of their low cost, but their low absorption rate and bioavailability increase the need for alternative iron sources. In this study, a nano-formed iron sulfur (nFeS), previously proved to have multivarious functions with high biocompatibility, is investigated as an iron supplement in broilers regarding its effects on growth performance, feed intake, feed conversion rate, systemic antioxidant status as represented by superoxide dismutase (SOD) and glutathione peroxidase (GSH-Px) activities, tissue iron deposition, breast meat quality as represented by color, postmortem pH, drip loss and shear force, and intestinal microbiota. A tolerance evaluation confirmed that at doses of up to 400 mg/kg, nFeS did not negatively impact broilers. Excessive iron was excreted within 3 days after completion of treatment. nFeS is an effective iron supplement with high bioavailability and comparability and it shows benefits in shaping intestinal microbiota.

## 1. Introduction

In modern fast-growing broilers, early life is characterized by rapid growth of body mass and a high metabolic rate; therefore, large amounts of iron are required by young animals [1]. An adequate iron supply during early growth is critical for broiler chickens. Iron serves as the core structural component of hemoglobin (Hb) and myoglobin and acts as an essential cofactor for multiple heme-containing enzymes involved in mitochondrial electron transport and cellular energy metabolism, which are particularly active during the early post-hatch period [2,3]. Insufficient iron intake not only impairs hematological parameters, such as hemoglobin concentration and packed cell volume (PCV), but also compromises the activities of iron-dependent enzymes in muscle and the intestinal epithelium, which limits growth performance and feed efficiency. Conversely, excessive iron supplementation may cause oxidative stress and interfere with the absorption of other trace minerals, through competition of mineral transporters. Therefore, an efficient and effective iron supply that meets the physiological requirements of rapidly growing broilers is of importance for overall productive performance.

Poultry diets typically rely on inorganic iron salts to meet iron requirements [4]. In June 2026, the *Gazette of the Ministry of Agriculture of P.R.C* updated several items on the list of inorganic iron sources that are allowed to supplement animal feed, including ferrous sulfate, citrate, chloride and carbonate. However, inorganic iron sources have several limitations. For example, they are prone to binding dietary phytate and fiber, thus reducing their intestinal absorption [5]. In addition, the rapid dissociation of ionic iron in the intestinal lumen can elevate ferrous ions, which can trigger the Fenton reaction and induce oxidative stress in the intestinal epithelia [6]. Therefore, developing alternative iron sources with improved bioavailability and biocompatibility is necessary in order to supply iron to animals while minimizing the risk of oxidative stress.

Recently, advances in nanotechnology have provided new strategies for mineral supplementation in animal nutrition. Nanominerals, characterized by their small particle size and large surface area, are considered to have the potential to improve mineral utilization efficiency [7]. Among these nanomaterials, nano-iron preparations have attracted increasing attention due to their nanoscale characteristics and unique physicochemical properties. Previous studies have demonstrated that nano-iron oxide supplementation could improve feed conversion ratio, jejunal morphology, and breast muscle morphology in broilers [8]. Meanwhile, nano-iron oxide has also been reported to improve growth performance, meat quality, and carcass traits in broilers [9]. In addition to nano-iron materials, other nanominerals, such as nano-zinc, nano-copper, and nano-selenium, have gradually been applied in poultry nutrition studies in recent years and have shown potential effects in promoting growth performance, regulating immune function, and improving antioxidant status [7]. The techniques of manufacturing nano-metals have significantly improved, and now allow a larger production scale. Thus, novel forms of nano-iron that are potentially efficient and safe for animals as feed supplements should be evaluated in vivo.

Nano-iron sulfide (nFeS) has attracted increasing attention as an iron-based biomaterial that is synthesized, inspired by decoction procedures of Traditional Chinese Medicine, using nano-iron oxide as the basis [9,10]. Unlike nano-iron oxide, nFeS is characterized by its ability to release iron ions in solution and by the presence of sulfur in its structure [11]. In aqueous media or simulated gastric fluid, the sulfur component can generate polysulfide species—including H_2_S_2_, H_2_S, and other higher polysulfides. These species can act as electron donors and form stable coordination bonds with transition metal ions. This is structurally analogous to the well-established chelation mechanism of amino acids, where carboxyl oxygen and amino nitrogen atoms coordinate with metal centers. It is widely accepted that organic minerals, particularly amino acid-chelated minerals, exhibit significantly higher absorption rates than their inorganic counterparts. Given the multiple merits, such as nanoscale dimensions, slow iron release, and the chemical stability conferred by its sulfur component, nFeS is likely to possess higher bioavailability than commonly used inorganic iron supplements. In our previous studies, nFeS has demonstrated multiple beneficial bioactivities, including antioxidant effects in cultured cells, antibacterial activity in vitro, protection of intestinal epithelial cells against ferroptosis, and enrichment of beneficial gut bacteria [12,13]. As a promising iron supplement, nFeS needs to be evaluated for its efficiency in supplying iron to broilers and for its optimal dose for growth performance.

Gut microbiota colonization is highly sensitive to luminal iron and redox status. nFeS may modify the redox status of the local intestinal environment because the unique sulfur components in nFeS, which are present as polysulfane in solution [11], can provide electrons as reductants. Iron deficiency is associated with dysbiosis of the intestinal microbiota and decreased levels of beneficial bacteria [14]. Iron supplementation can reshape and help maintain the balance of the intestinal microbial community [15]. In addition, iron-associated changes in the gut microbiota are related to oxidative status and lipid metabolism [16]. These facts suggest that iron, gut microbiota, growth performance, and meat quality are closely interconnected in broilers [17,18].

Therefore, in this study we investigated the roles of nFeS as an iron source in systemic biological functions in broilers by evaluation of growth performance, iron status in tissues, antioxidant status, breast meat quality, lipid metabolism, organ indices, and gut (jejunal and cecal) bacterial composition. In addition, we tested the tolerance of broilers to a higher dose of nFeS (up to 400 mg/kg) by measuring biomarkers of tissue function and tissue iron deposition, and by conducting histopathological examinations of representative tissues. This study aimed to evaluate the efficacy of nFeS as an iron source for supplementation in broiler feed.

## 2. Materials and Methods

### 2.1. Ethical Statement

This experiment was carried out at the National Feed Engineering Technology Research Center of the Ministry of Agriculture Feed Industry Center Animal Testing Base (Hebei, China). All procedures used in this study were conducted in accordance with the Chinese Guidelines for Animal Welfare and approved by the China Agricultural University Institutional Animal Care and Use Committee (Approval No. VW59769823).

### 2.2. nFeS Source

The nFeS (nano Fe_3_S_4_) nanoparticles (99.9% purity; average particle size 50~200 nm) used in this study were synthesized using the hydrothermal synthesis method previously described [11]. The morphology, crystal structure, and particle size distribution of the nFeS prepared using this method have been characterized by transmission electron microscopy (TEM; FEI Tecnai G2 F30, FEI Company, Hillsboro, OR, USA), X-ray diffraction (XRD; D8 ADVANCE, Bruker Corporation, Billerica, MA, USA; analyzed using Jade 6.5, MDI, Livermore, CA, USA), and particle-size analysis (in our previous study) [13]. Briefly, FeCl_3_ (0.82 g) was dissolved in 40 mL of ethylene glycol by stirring (30 min) and ultrasonication (10 min). NaAc·3H_2_O (3.6 g) was then added and stirred for 30 min, followed by dropwise addition of 1 mL of diallyl disulfide under vigorous stirring for 30 min. The mixture was transferred to a 50 mL Teflon-lined autoclave and heated at 200 °C for 12 h. After cooling, the black precipitate was washed with water and ethanol (6 cycles) and vacuum-dried at 60 °C for 5 h.

### 2.3. Animals, Treatments, and Diet Formulation

To evaluate the efficacy of nFeS as an iron source in broilers, a total of 180 healthy 1-day-old male Arbor Acres (AA) broilers (initial body weight 43 ± 0.4 g) were randomly assigned to five dietary treatments (6 replicates/treatment, 6 broilers/replicate). The treatment lasted for 42 days. The five dietary treatments were as follows. Iron-deficient diet (ID): basal diet formulated according to NRC (1994) requirements, except no supplemental iron—only background iron (Table 1). Control diet (CON): ID supplemented with 80 mg/kg Fe as ferrous sulfate (FeSO_4_·H_2_O). nFeS-supplemented diets (L-, M-, H-FeS): ID supplemented with 20, 40, or 80 mg/kg Fe as nFeS. All diets were formulated according to NRC (1994), with iron as the only experimental variable. Dietary iron levels were expressed as elemental Fe. The basal diet contained 35–38 mg/kg Fe. In CON, FeSO_4_·H_2_O was included at 243.4 mg/kg diet to provide 80 mg/kg elemental Fe. In L-, M-, H-FeS, nano-ferrous sulfide (nFeS) was included at 50, 100, and 200 mg/kg diet to provide 20, 40, and 80 mg/kg elemental Fe, respectively. Broilers were housed in climate-controlled cages with ad libitum access to feed and water provided through trough feeders and nipple drinkers. The lighting schedule was maintained at 23 h light and 1 h darkness during the first week and adjusted to 20 h light and 4 h darkness from day 8 until the end of the experiment. The house temperature was set at 33 °C initially, then reduced by 3 °C weekly until reaching 24 °C on day 21, where it remained for the rest of the trial. Relative humidity was maintained between 50% and 70% throughout the trial, and a negative-pressure ventilation system was used to maintain proper air exchange.

For the tolerance study of nFeS, broilers were treated with higher doses of iron supplement. In sum, 108 1-day-old male Arbor Acres broilers (43 ± 0.5 g) were randomly allocated to three dietary treatments (6 replicates/treatment, 6 broilers/replicate) for a 42-day trial. The three diets were Standard diet (SS), S200 and S400, respectively. SS was set as the control, which was the basal diet with 243.4 mg/kg FeSO_4_·H_2_O to provide 80 mg/kg Fe, as NRC (1994) recommended. S200 and S400 were SS supplemented with 200 or 400 mg/kg nFeS to provide 160 (80 mg/kg Fe from SS FeSO_4_ and 80 mg/kg Fe from nFeS) or 240 mg/kg Fe (80 mg/kg Fe from SS FeSO_4_ and 160 mg/kg Fe from nFeS) as the treatments. The housing and management conditions were identical to those described above.

Feed crude protein, calcium, and total phosphorus were analyzed according to the procedures defined by the Association of Official Analytical Chemists (AOAC 2016 [19]), and iron concentrations were measured using inductively coupled plasma optical emission spectrometry (ICP-OES; Avio 200, PerkinElmer, Waltham, MA, USA) at an analytical wavelength of 238.204 nm.

Body weights and feed consumption were recorded on a pen basis on days 1, 21, and 42 to calculate average daily gain (ADG), average daily feed intake (ADFI), and feed conversion ratio (FCR). On days 21 and 42, one broiler per replicate (with a body weight close to the replicate average) was selected for wing vein blood collection and tissue sampling. The spleen, thymus, and bursa of Fabricius were collected and weighed, and the relative immune organ indices were calculated as organ weight (g) per kilogram of live body weight (g/kg). On day 42, broilers were slaughtered, immediately after a 12 h feed withdrawal, for blood, tissue, and intestinal digesta sampling. Specifically, in the nFeS tolerance evaluation, 3 broilers per replicate were randomly selected for slaughter according to the above procedure, while the remaining 3 broilers per replicate were transferred to individual metabolism cages and switched to the standard diet (SS). Fecal samples within each replicate were pooled daily (days 43–49) for iron clearance analysis.

### 2.4. Measurement of Tissue Iron and Serum Biochemical Indices

Tissue iron concentrations were measured after acid digestion, using ICP-OES according to previously described methods [20]. Iron concentrations in duodenal, jejunal and cecal digesta were determined after acid digestion, using atomic absorption spectrometry (AAS; AA-6880, Shimadzu Corporation, Kyoto, Japan) according to a previously described method [21]. Serum iron concentrations were determined using a commercial ferrozine-based colorimetric assay kit (A039-1-1, Nanjing Jiancheng Bioengineering Institute, Nanjing, China) according to the manufacturer’s instructions. To further evaluate broiler tolerance under higher doses, serum samples were also analyzed for extended iron status (ferritin and total iron-binding capacity, TIBC), hepatic function (ALT, AST, TP, ALB, and T-BIL), and renal function (BUN and CREA) using an Olympus AU480 automated biochemical analyzer (Olympus, Tokyo, Japan).

Lipid profiles (TC, TG, HDL-C, and LDL-C) and antioxidant status (SOD, GSH-Px, and MDA, measured on day 42) were determined using commercial kits (Nanjing Jiancheng Bioengineering Institute, Nanjing, China) following the manufacturer’s instructions. For the tolerance analysis, extended serum antioxidant capacity (CAT and T-AOC) was assessed similarly. Hemoglobin (Hb) and packed cell volume (PCV) were analyzed via an automated hematology analyzer (BC-2800VET, Mindray, Shenzhen, China). Whole blood from the tolerance evaluation was further analyzed for extended hematological parameters (including WBC counts, MCV, MCH, and PLT) using the same automated hematology analyzer.

### 2.5. Measurement of Meat Quality

Meat quality was assessed as previously described [22]. Briefly, muscle pH at 45 min and 24 h after slaughter (pH_45min_ and pH_24h_) was determined in triplicate using a portable pH meter equipped with a glass insertion electrode (Testo 205; Testo SE & Co. KGaA, Titisee-Neustadt, Germany). Lightness (L*), redness (a*), and yellowness (b*) values were determined at 24 h post-mortem using a chromameter (CR-400; Konica Minolta, Tokyo, Japan). Shear force was measured using an Instron 4411 (Instron Corporation, Norwood, MA, USA). The water-holding capacity (WHC) of meat was evaluated by determining the drip loss as previously described. Every measurement was performed in triplicate.

### 2.6. Analysis of Gut Microbiota

Total microbial genomic DNA was extracted from digesta using the E.Z.N.A. Stool DNA Kit (Omega Bio-tek, Norcross, GA, USA) according to the manufacturer's instructions and the V3–V4 regions of the bacterial 16S rRNA gene were amplified using the universal primers 338F (5′-ACTCCTACGGGAGGCAGCAG-3′) and 806R (5′-GGACTACHVGGGTWTCTAAT-3′). Amplicons were sequenced on the Illumina MiSeq/NovaSeq platform (Biozeron, Shanghai, China). Sequence processing, taxonomic annotation based on the Silva database (v138), and microbial community analyses were performed using the Majorbio Cloud Platform (http://www.majorbio.com).

Alpha diversity was evaluated using the Chao1 and Shannon indices, and differences among groups were analyzed using the Kruskal–Wallis test with Tukey–Kramer post-hoc comparisons after FDR correction. Beta diversity was assessed by principal coordinate analysis (PCoA) based on abundance-based Jaccard distances, and microbial community differences were evaluated using ANOSIM with 999 permutations. Taxonomic composition was summarized at the genus level, with genera showing relative abundances below 1% merged into “others”. Differential bacterial taxa were identified using Kruskal–Wallis analysis with FDR correction and visualized using extended error bar plots.

### 2.7. Histopathological Examination

Tissues were fixed in 4% paraformaldehyde, paraffin-embedded, and sectioned at 5 μm. Sections were stained with hematoxylin and eosin (H/E) to assess morphological changes and inflammatory infiltration. Prussian blue staining was performed to quantify iron deposition using an image analysis system (BX53, Olympus, Tokyo, Japan).

### 2.8. Statistical Analysis

All data were analyzed by one-way analysis of variance (ANOVA) using SPSS version 26.0 (IBM Corp., Armonk, NY, USA) with the replicate pen defined as the experimental unit. Significant differences among treatments were determined using Tukey’s multiple comparison test. Results are expressed as the mean and standard error of the mean (SEM). Statistical significance was defined as *p* < 0.05, and a trend toward significance was considered at 0.05 ≤ *p* < 0.10.

## 3. Results

### 3.1. Effects of nFeS on Growth, Systemic Health, and Gut Microbiota

#### 3.1.1. Growth Performance and Immune Organ Indices

**Figure 1:**
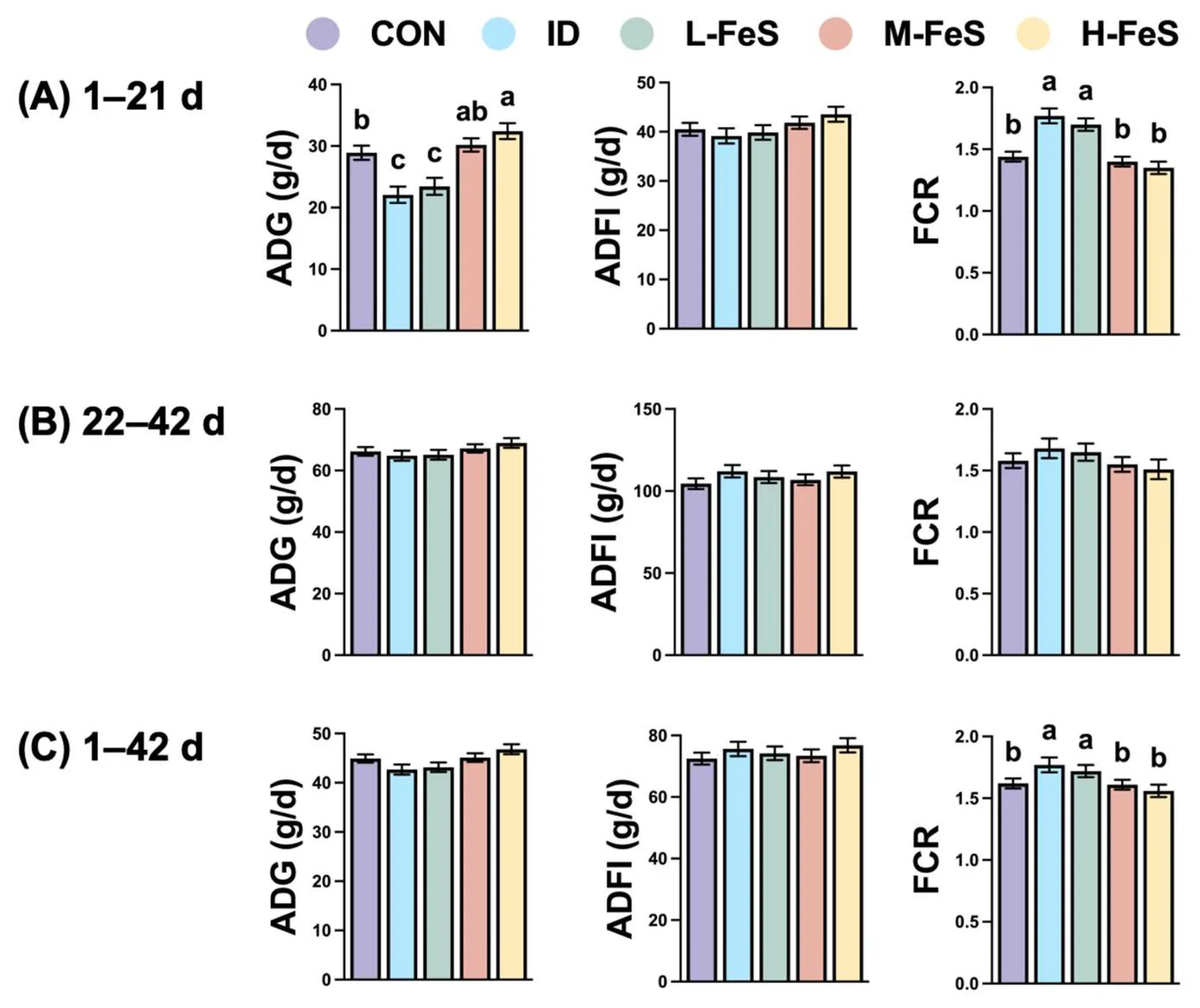
nFeS effects on growth performance of AA broilers: (**A**) growth performance during the starter period (1–21 d), including average daily gain (ADG), average daily feed intake (ADFI), and feed conversion ratio (FCR); (**B**) growth performance during the grower period (22–42 d); and (**C**) overall growth performance during the entire experimental period (1–42 d). Values are presented as means with their standard errors (SEM). Means within a chart with different superscript letters (a–c) differ significantly (*p* < 0.05).

nFeS supplementation significantly improved ADG and FCR in the M-FeS and H-FeS groups compared with the ID group during the starter phase (days 1–21) (*p* < 0.01), whereas ADFI was not significantly altered (*p* = 0.185) compared with the ID groups (Figure 1A). Notably, H-FeS showed a significantly higher ADG than the CON group during the starter phase (days 1–21) (*p* < 0.05). The effects of H-FeS were comparable to or even greater than those of FeSO_4_ groups. In the grower phase (days 22–42), nFeS effects were similar to those of FeSO_4_ regarding ADG, ADFI and FCR, which indicated broiler growth in the starting period was more sensitive to the available irons (Figure 1B). Over the entire experimental phase (days 1–42), compared with ID, M- and H-FeS showed improved FCR, as shown in the starter phase (*p* < 0.05) (Figure 1C).

Dietary treatments did not significantly affect the spleen, thymus, or bursa of Fabricius indices on days 21 and 42 (*p* > 0.05). However, nFeS supplementation tended to increase the bursa of Fabricius index on day 42 (*p* = 0.082), with broilers in the M-FeS and H-FeS groups showing numerically higher values than those in the ID group (Table S2).

#### 3.1.2. Hematological and Serum Biochemical Parameters

Concerning the enhancement of hemoglobin, nFeS supplementation significantly increased the whole-blood content of Hb and the percentage of PCV, compared with the ID group (Table 2). This effect was observed during the starter phase (days 1–21) (*p* < 0.001). During this phase, the H-FeS group, which had a dietary Fe level similar to the FeSO_4_ supplementation group, exhibited the significantly superior effects of elevated Hb and PCV levels. These results demonstrated that nFeS performed more effectively than inorganic Fe in the form of FeSO_4_ did in maintaining whole-blood Hb content during the early growth stage. During the grower phase (days 22–42), the M- and H-FeS groups showed numerically higher whole-blood Hb content and PCV percentage relative to the CON group.

However, serum lipid indices, including TC, TG, HDL-C, and LDL-C concentrations, were not significantly affected by either nFeS or FeSO_4_ at the current doses in both phases. Serum redox indices were significantly affected by nFeS treatment on day 42. The treatment of H-FeS had optimal performance as to increasing the activities of SOD and GSH-Px. Concurrently, compared with the ID group, the M- and H-FeS groups significantly increased the activities of SOD and GSH-Px (*p* < 0.05). Moreover, H-FeS exhibited significantly higher SOD and GSH-Px activities than the CON group. Furthermore, the MDA levels indicated that dietary nFeS supplementation could significantly decrease oxidative damage in broilers (*p* < 0.05).

#### 3.1.3. Iron Status and Tissue Iron Deposition

On days 21 and 42, nFeS supplementation significantly increased serum iron concentrations in the M-FeS and H-FeS groups compared with the ID group (*p* < 0.05). On day 42, dietary nFeS treatments had significantly higher tissue iron content in the liver, spleen, and duodenum (*p* < 0.05, Table 3). The iron content in jejunal and cecal digesta on day 42 of the nFeS treatment groups was significantly lower than that of CON (*p* < 0.05). These results suggest that nFeS may improve intestinal iron utilization compared with FeSO_4_, as evidenced by the lower iron content observed in the digesta of the jejunum and cecum.

#### 3.1.4. Meat Quality

Regarding meat color, the M-FeS and H-FeS groups exhibited significantly higher L* and a* values of breast muscle compared with the ID group (*p* < 0.05, Table 4). Specifically, the L* values of the M- and H-FeS groups were significantly higher than those of the CON group, and the a* values also exhibited a numerical advantage over the FeSO_4_ treatment group. No significant differences were observed in yellowness (b*) values among all groups.

In terms of muscle pH, the M- and H-FeS groups significantly elevated the breast pH at 45 min post-mortem compared with the ID group (*p* < 0.05), with effects comparable to the FeSO_4_ treatment. Conversely, the breast pH at 24 h and the thigh pH at all measured time points remained relatively stable across all groups. Compared with the ID group, the M- and H-FeS groups significantly decreased the drip loss of breast muscle (*p* < 0.05) and reached a level comparable to the FeSO_4_ treatment, suggesting that nFeS supplementation alleviated the increased drip loss induced by iron deficiency. Additionally, no significant difference was detected in muscle shear force among the treatment groups (*p* = 0.462).

Dietary supplementation with nFeS or FeSO_4_ exerted no significant effects on the slaughter performance of broilers, including breast muscle yield (*p* = 0.662) and thigh muscle yield (*p* = 0.241).

#### 3.1.5. Intestinal Microbial Community Structure and Spatial Dispersal Characteristics

**Figure 2:**
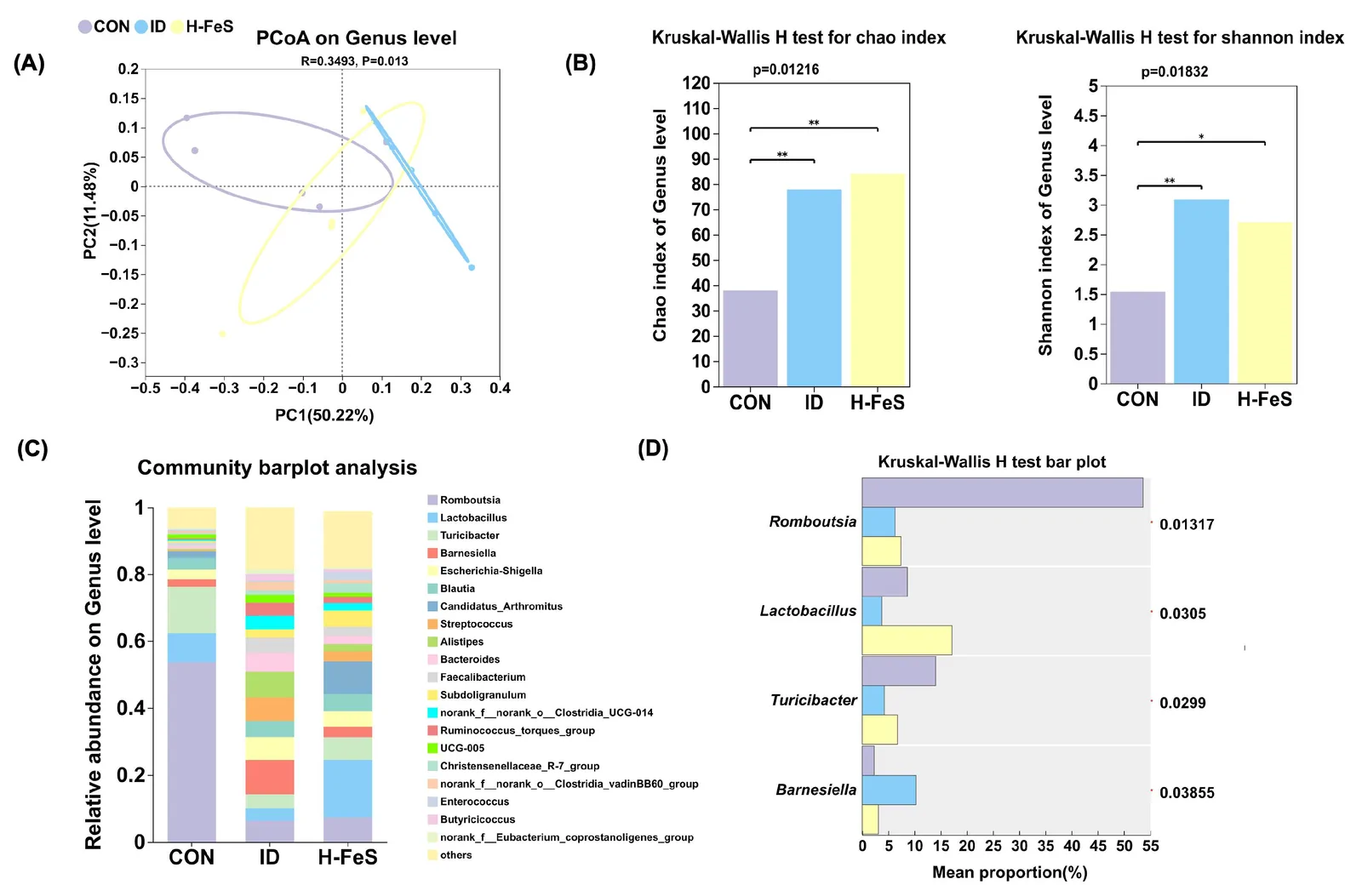

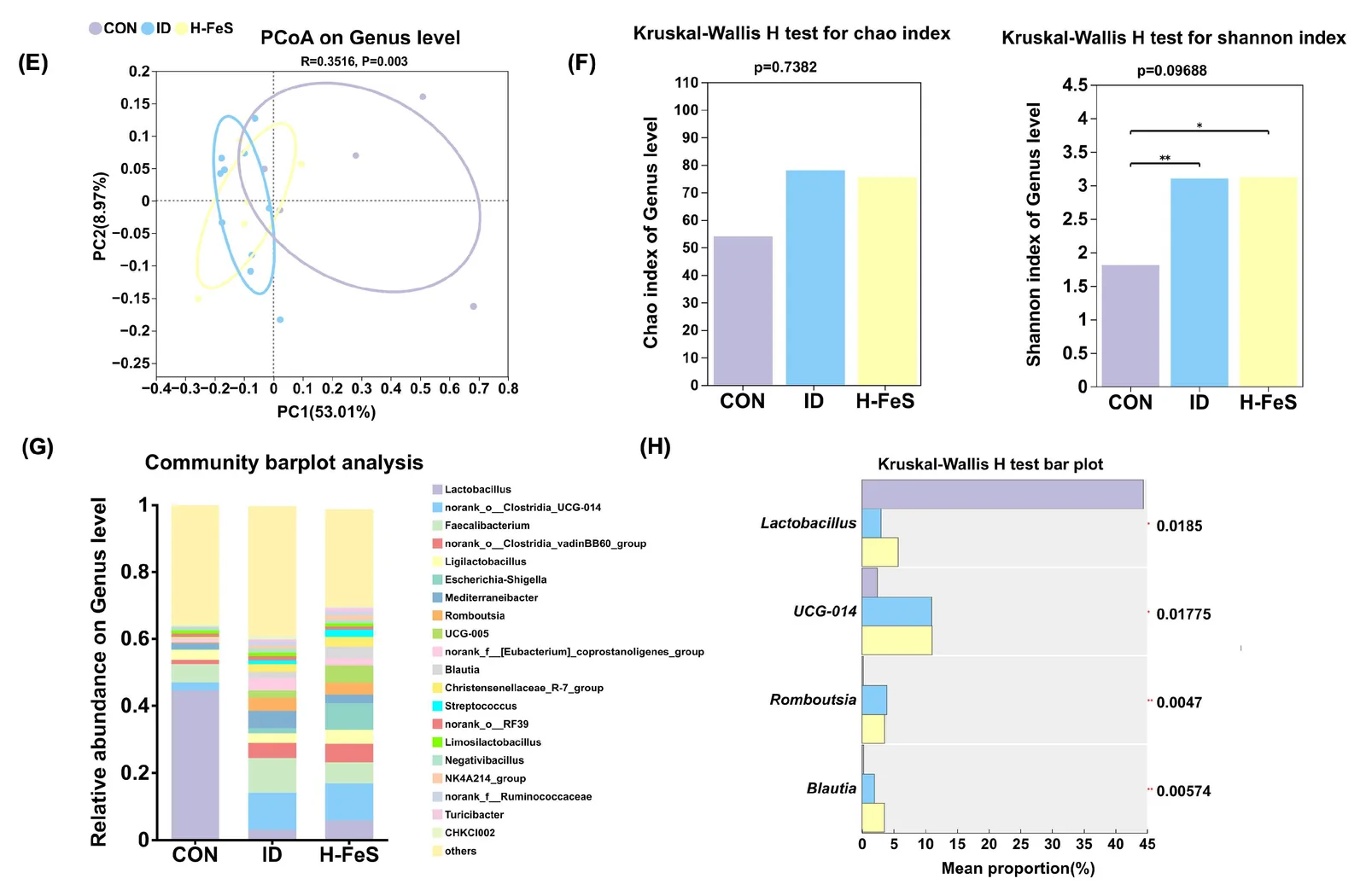
Effects of nFeS on jejunal and cecal microbiota of AA broilers. (**A**–**D**) Jejunal microbiota analysis: (**A**) principal coordinate analysis (PCoA) on the genus level; (**B**) alpha diversity indices (Chao and Shannon) at the genus level; (**C**) community barplot analysis illustrating the relative abundance on the genus level; and (**D**) differential genus analysis using Kruskal–Wallis H test bar plot. (**E**–**H**) Cecal microbiota analysis: (**E**) PCoA on the genus level; (**F**) alpha diversity indices (Chao and Shannon) at the genus level; (**G**) community barplot analysis on the genus level; and (**H**) differential genus analysis using Kruskal–Wallis H test bar plot. Significant differences between groups are indicated by asterisks (0.01 < * *p* ≤ 0.05, 0.001 < ** *p* ≤ 0.01).

Iron deficiency caused profound alterations in the microbial compositions of both the jejunum and cecum of broilers (Figure 2). In the jejunum, both Chao and Shannon indices were significantly higher in the H-FeS group compared with the CON group (*p* < 0.05). In the cecum, no significant differences were observed in the Chao index among groups, whereas the Shannon index tended to increase in the H-FeS group compared with the CON group. Beta diversity analysis (PCoA) revealed that the microbial community structures in both the jejunum (*R* = 0.3493, *p* = 0.013) and the cecum (*R* = 0.3516, *p* = 0.003) were significantly altered by Fe treatment, with each group displaying distinct spatial separation.

At the genus level, compared with the ID group, nFeS treatments significantly increased the relative abundances of *Lactobacillus* in the jejunum (*p* < 0.05), while reducing the abundance of bacteria such as *Alistipes*, *Barnesiella*, and *Bacteroides*. Notably, the enrichment of *Lactobacillus* in the nFeS treatment was much more pronounced than that of FeSO_4_ treatment, suggesting that nFeS performed more effectively in enrichment of the beneficial bacteria represented by *Lactobacillus* than FeSO_4_.

In the cecum, the H-FeS treatments exhibited a numerically higher relative abundance of *Lactobacillus* compared with the ID treatment; however, regarding the overall bacterial composition, the profile of the nFeS-treated group remained highly similar to that of ID. This indicated that nFeS was absorbed rapidly in the jejunum (proximal small intestine), while the residual iron reaching the cecum exerted only a marginal influence on the microbial community. Consequently, the nFeS group exhibited a typical iron-deficient bacterial profile, similar to that of the ID group.

### 3.2. Tolerance of nFeS Supplementation in Broilers

The S200 and S400 groups showed no significant effects on the ADG, ADFI, or FCR of broilers (days 1–42) (*p* > 0.05, Table 5). Consistently, the whole-blood content of Hb and the percentage of PCV showed no significant differences among dietary treatments on days 21 and 42 (*p* > 0.05). Furthermore, other hematological parameters, including MCV, MCH, WBC, and PLT, were not significantly altered in the S200 and S400 groups compared with the SS group (*p* > 0.05, Table 6).

**Figure 3:**
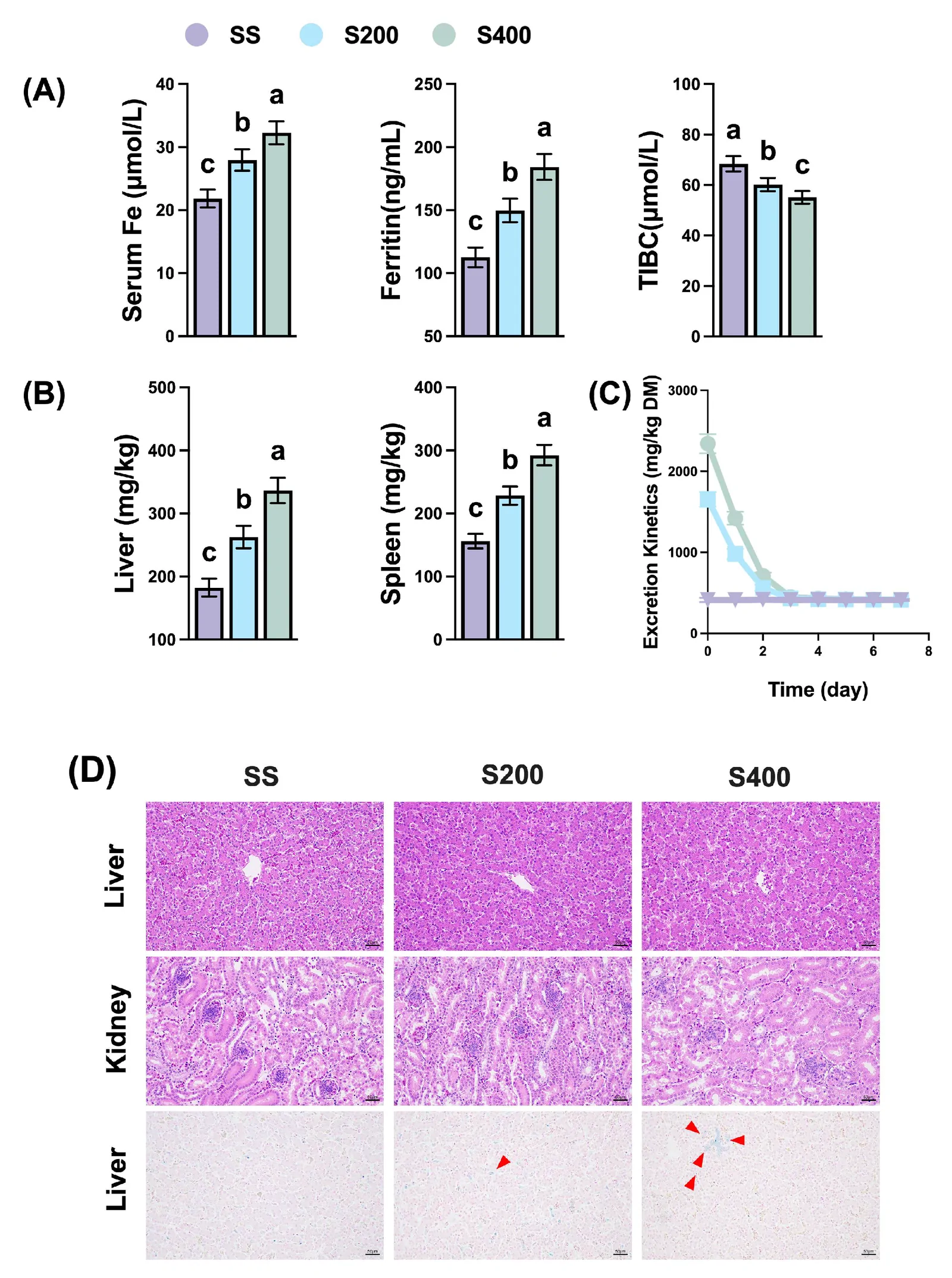
Tolerance evaluation of high-dose dietary nFeS and withdrawal in AA broilers: (**A**) iron parameters, including serum iron (Serum Fe), serum ferritin, and total iron-binding capacity (TIBC) on day 42; (**B**) tissue iron content in the liver and spleen on day 42; (**C**) dynamic post-exposure fecal iron clearance kinetics from day 42 to day 49 (1–7 days post-withdrawal); and (**D**) representative histological micrographs of liver and kidney tissues on day 42. The top two rows show hematoxylin and eosin (H/E)-stained sections displaying normal architecture. The bottom row shows Prussian blue-stained liver sections, with red arrowheads indicating stained iron. Scale bar = 50 μm (200× magnification). Means within a panel with different letters (a–c) differ significantly (*p* < 0.05) (*n* = 6).

The S200 and S400 groups caused significantly higher serum iron concentrations and ferritin levels (*p* < 0.05) compared to SS, whereas TIBC was significantly lower (*p* < 0.05, Figure 3A). Increased doses of dietary nFeS enhanced the tissue iron deposition in the liver and spleen (*p* < 0.05, Figure 3B). Dynamic excretion kinetics analysis revealed that following the transition of nFeS treatment to the standard basal diet on day 42, fecal iron excretion in the S200 and S400 groups returned to baseline levels within approximately 3 days post-withdrawal, relative to the SS group (Figure 3C).

Representative H/E-stained sections of the liver and kidney showed normal histological architecture and cellular morphology in the S200 and S400 groups, which were comparable to those of SS, with no inflammatory cell infiltration or pathological lesions observed. Prussian blue staining demonstrated higher levels of iron-positive granules in the livers (Figure 3D).

Compared with SS, S200 and S400 showed no significant effects on serum ALT, AST, T-BIL, TP, ALB, BUN, or CREA (*p* > 0.05, Table 7). Similarly, systemic antioxidant parameters and the relative weights of the liver, spleen, and kidney were not significantly affected among the treatments (*p* > 0.05, Table 7 and Table 8).

## 4. Discussion

As a micronutrient, iron is essential for animal survival. For broilers, the dietary iron requirement recommended by NRC (1994) is approximately 80 mg/kg of diet to support normal growth performance and physiological functions. Inorganic iron has a low absorption rate. In humans, the bioavailability of inorganic iron (non-heme) is about 2 to 15%, compared to 10 to 25% for heme iron [23]. In addition, the absorption of inorganic iron is inhibited by numerous molecules such as phytic acid, polyphenols, calcium, oxalate, and whey protein [24]. Comparing ferric (Fe^3+^) and ferrous (Fe^2+^) ions, ferric ions have a higher affinity for these molecules than ferrous ions do. At intestinal pH, ferric ions readily reduced the concentrations of bioavailable iron because they precipitate as insoluble ferric hydroxides [25]. Dietary ferric ions need to be converted to ferrous ions before being absorbed by enterocytes via divalent metal transporter 1 (DMT1). The duodenal and jejunal enterocytes are the primary sites of iron absorption, where duodenal cytochrome b (DcytB) and other brush border ferrireductase catalyze the reduction of Fe^3+^ to Fe^2+^.

The absorbed Fe^2+^ ions in enterocytes undergo one of two fates, either export or storage, depending on systemic iron demand. When systemic iron stores are depleted, absorbed iron is transported across the basolateral membrane of enterocytes via the iron export protein ferroportin. Subsequently serum transferrin delivers the iron to tissues mediated by the ferroxidase located on the basolateral membrane, which converts Fe^2+^ to Fe^3+^ before the iron is bound to transferrin [26,27]. The functional rationale for the redox cycling between Fe^2+^ and Fe^3+^ is to sequester ferrous iron to protect against oxidative cellular damage. Ferrous iron is unstable and easily oxidized to ferric iron (Fe^3+^), during the reaction; highly reactive hydroxyl radicals (·OH) are produced via the Fenton reaction, which can damage macromolecules within cells. Thus, ferrous iron cannot exist in its free form. It must be bound to small molecules such as reductants (e.g., NADH, NADPH, ascorbic acid, and glutathione [28]), chaperones (e.g., PCBP1 [29]), or phosphates and citrate [30]. The tissues that need iron acquire iron from serum transferrin via the transferrin receptor expressed on the cell surface. Transferrin is internalized via receptor-mediated endocytosis, during which Fe^3+^ is released from transferrin from the carrier proteins within acidified endosomes. Within these cells, ferric iron is either reduced to ferrous iron for cellular utilization or stored in ferritin.

When body iron stores are sufficient, ferrous iron taken up by the enterocyte can be incorporated into the ferritin core, where it is stored as ferric ion (Fe^3+^). Notably, the ferritin heavy chain is a ferroxidase that converts Fe^2+^ to Fe^3+^. The storage of iron in enterocytes is short-term, as enterocytes are short-lived. They are shed from the villus tip within 1–2 days. The iron not transported out of enterocytes during this time is lost in the lumen. The iron not incorporated into ferritin is stored in the cytosolic labile iron pool (LIP). This iron is predominantly in the ferrous form; it is bound to small molecules and can be readily exported via ferroportin and subsequently oxidized to Fe^3+^ by ferroxidase before binding to transferrin, a process catalyzed by ferroxidase. In contrast, the liver and spleen are the major sites for both long-term iron storage and iron recycling from senescent erythrocytes.

In recent years, nanotechnology has developed rapidly, and it has attracted considerable interest from animal scientists. The nanoparticles, typically with a size of less than 100 nm, may enhance the absorption and bioavailability of iron in chickens [31]. For example, nano-iron oxide supplemented at 60 mg/kg in the diet resulted in higher gain-to-feed ratio (G:F) compared with ferrous sulfate [8]. In the current study, the effects of nFeS supplementation providing dietary iron at levels ranging from 20 to 80 mg/kg were investigated in broilers. The results showed improved feed efficiency, increased blood hemoglobin concentration at the early growth stage, and higher iron content in the tissues (serum, liver and spleen), indicating improved absorption and bioavailability of nFeS compared with FeSO_4_. In this study, nFeS supplementation increased hemoglobin concentration during the starter phase, but had no significant effect during the grower phase. This stage-dependent alteration may be related to the regulation of iron homeostasis. Iron is allocated according to physiological requirements [27]. In the starter phase, the increased hemoglobin concentration at day 21 suggested that nFeS could effectively support erythropoiesis. As iron supplementation continued, hemoglobin concentration remained relatively stable during the grower phase, indicating that the iron requirement for hemoglobin synthesis had been largely met. Meanwhile, the additional absorbed iron may have been directed toward iron storage, which may be reflected by the increased tissue iron concentrations in nFeS-supplemented groups. Together with the improved tissue iron status and hematological parameters, these findings further support the determination that nFeS exhibits high iron bioavailability compared with FeSO_4_ and can effectively maintain iron homeostasis in broilers.

These results were attributed to the structural characteristics and chemical composition of nFeS. nFeS is synthesized from FeCl_3_ and garlic-derived organosulfur compounds, and other natural organosulfur sources, using solvothermal methods to form nanosheet-like hexagonal structures [11]. The structure is different from those of typical nanoparticles such as Fe_3_O_4_, but it has been shown that nFeS slowly releases predominantly Fe^2+^ (93%), along with small amounts of Fe^3+^ and polysulfide species (mixed forms of H_2_S_2_, H_2_S and other polysulfide species), in aqueous solutions or simulated gastric acid fluid [32]. The sulfur component in nFeS can stabilize ferrous ions by continuously providing electrons to ferric ions (Fe^3+^) and reducing Fe^3+^ back to Fe^2+^, thereby stabilizing the ferrous state and enhancing its bioavailability. [33]. Additionally, the nanosheet structure of nFeS is gradually dissolved and decomposed into nanoparticles with the sizes ranging from 20 to 50 nm [13,32]. This release characteristic of nFeS allows both ionized iron and nanosized nFeS to exist in the solution or the intestinal lumen. The mechanisms of nanoparticle uptake by mammalian cells include phagocytosis, micropinocytosis, clathrin-mediated endocytosis and caveolae-mediated endocytosis, depending on particle size and cell type [34]. For intestinal epithelial cells, nanoparticles are taken up via endocytosis at the apical sides of enterocytes [35]. The uptake of nFeS as a particle rather than pure released ions has been observed in *Lactobacillus* [13], and indirectly demonstrated in macrophages [33], although no studies have been conducted on intestinal epithelial cells. Therefore, nFeS is believed to be taken up by broiler tissue cells via divalent metal transporters for ferrous iron transport, and via endocytosis for intact particle internalization of nFeS, which may explain the higher absorption and bioavailability of nFeS compared with FeSO_4_. This conclusion is further supported by the relationship between nFeS and *Lactobacillus* abundance in the intestines. Our previous study revealed that nFeS was biocompatible with *Lactobacillus* strains [13] and could enrich *Lactobacillus* in pig gut. In the present study, we found a low abundance of *Lactobacillus* in nFeS-treated broilers, in the cecum. This microbial distribution profile was similar to that in the iron-deficient group (Figure 2). This result indicated that nFeS was absorbed to a greater extent in the proximal small intestine relative to FeSO_4_, and that much less nFeS remained in the gut. In contrast, in the jejunum, nFeS treatment resulted in a higher abundance of *Lactobacillus* than in FeSO_4_. This hypothesis was further supported by the measurement of iron content in duodenal, jejunal and cecal digesta (Table 3).

Traditional metal oxide nanoparticles are usually characterized by producing ROS via the Fenton reaction, which underlies their antibacterial activity [36]. In contrast, several newly developed nanometal materials are capable of scavenging free radicals [37,38]. Iron-based nanomaterials have high biocompatibility for biomedical applications. Iron oxide nanoparticles have been approved by the US Food and Drug Administration (FDA) for the clinical treatment of iron deficiency anemia and ferroportin-deficient leukemia [39,40]. Previous studies have revealed that nFeS exhibits a broad spectrum of activity against Gram-positive and Gram-negative bacteria [11] and influenza virus [41], and can eliminate biofilm and intracellular bacteria [42,43]. nFeS induces ferroptosis in bacteria by releasing Fe^2+^ and depleting GSH, thereby exacerbating the oxidative damage. However, ferroptosis in bacteria is distinct from eukaryotic ferroptosis, because eukaryotes possess various regulatory components and endogenous anti-oxidative enzymes. These regulatory components and anti-oxidative enzymes, which are absent in bacteria, help protect eukaryotic cells from ferroptosis under normal conditions, whereas bacteria are more susceptible to ferroptosis, due to the lack of such defense mechanisms. [44]. Previous studies using doses higher than the physiological requirement in pigs (100 to 500 mg/kg) and in mice (1 g/kg) found up-regulation of intestinal epithelial FTH-1 and GPX-4 [13], and cortex SOD and CAT [32]. These results demonstrated the in vivo antioxidant effect of nFeS. In the present study, we used nFeS at doses ranging from 20 to 80 mg/kg, which were at or below the iron requirement listed in NRC (1994). And the nFeS improved antioxidant status, as represented by higher activity levels of serum SOD and GSH-PX, and the lower levels of MDA in broilers. Collectively, the antioxidant effects of nFeS have been demonstrated in pigs, mice and broilers. The higher antioxidant levels induced by nFeS may be attributed to ROS generated by Fe^2+^ released from nFeS, which could activate the endogenous defense system. Several potential pathways may explain the antioxidant effect of nFeS in animals. Firstly, previous studies have suggested that nFeS modifies the Nrf2-binding protein Keap1 via S-persulfidation of cysteine residues in Keap1. This modification induces a conformational change in Keap1, leading to the release of Nrf2 from the cytoplasm to the nucleus. Nrf2 masters cellular stress response in response to disrupted redox balance [45]. Its activation upregulates numerous genes involved in the response to cellular oxidative stress [32]. In addition, nFeS has been reported to maintain intracellular GSH levels by inhibiting GSH-degrading enzymes [46]. Another potential mechanism is that nFeS may upregulate the heavy chain of ferritin 1 (FTH-1) to sequester intracellular Fe^2+^ [13] and limit the Fenton reaction [47]. Additionally, transcriptome assays have revealed that nFeS upregulates the expression of heme oxygenase-1 [33] and iron-sulfur cluster family proteins [13]. Previous studies have suggested that oxidative stress is associated with myoglobin oxidation and meat discoloration. Excessively oxidative conditions may accelerate myoglobin oxidation and increase metmyoglobin formation, resulting in reduced meat redness [48,49]. In the present study, nFeS supplementation improved antioxidant status, as indicated by increased SOD and GSH-Px activities and decreased MDA levels. Therefore, the enhanced antioxidant capacity induced by nFeS may help alleviate ROS-mediated myoglobin oxidation and maintain myoglobin in favorable reduced or oxygenated states, which may contribute to the increased a* value observed in this study.

Iron is essential for fundamental cellular and organ functions. However, iron overload can lead to cell damage, primarily via ferroptosis. To evaluate the tolerance of broilers to nFeS, dietary supplementation with 400 mg/kg nFeS was provided to broilers. The results are consistent with our previous studies [13], which showed that mammals are tolerant to dietary nFeS up to 400 mg/kg in mice and 500 mg/kg in piglets. In these animals, the organs of liver, kidney, and intestines showed normal histology and serum metabolic markers. Excess iron was excreted via feces to a basal level in 7 days in mice and 14 days in piglets. In cultured intestinal epithelial cells and hepatocytes, a concentration of up to 1024 mM of nFeS had no negative effect on cell viability [33]. In this study, broilers treated with a high dose of nFeS up to 400 mg/kg showed no significant differences, compared with those receiving with normal supplementation, in growth performance, liver function markers, and relevant metabolic indices. Indeed, the high dose of nFeS was designed based on ferrous content rather than sulfur content, because broilers are relatively tolerant to dietary sulfur at levels of up to 4000 mg/kg, according to NRC (2005). Taken together, animals such as broilers, pigs and mice are tolerant to nFeS in the range of 400 to 500 mg/kg without adverse effects on metabolic indices.

As a novel iron source, nFeS has potential applications in commercial broiler production. The present study demonstrated that nFeS improved iron utilization and physiological status in broilers, supporting its application as an efficient iron source for poultry nutrition. Therefore, compared with the conventional inorganic iron source FeSO_4_, nFeS may improve iron supplementation efficiency in broiler production. Currently, studies on nFeS are mainly conducted under experimental conditions, and further investigations are needed to evaluate its long-term effects under commercial production conditions. Overall, nFeS has potential as an efficient iron source in poultry nutrition, and further optimization of the production process and systematic evaluations are required before its large-scale application.

## 5. Conclusions

Nano-ferrous sulfide (nFeS) effectively alleviated iron deficiency in broilers by improving early growth performance, restoring hematological status, and enhancing systemic antioxidant capacity. In addition, dietary nFeS improved breast meat quality and modulated intestinal microbial composition, with more pronounced effects observed in the jejunum. Furthermore, high-dose dietary nFeS supplementation was well tolerated without adverse effects on growth performance, hepatorenal function, or tissue histopathology. Collectively, these findings demonstrate that nFeS is an effective iron source with high bioavailability and biocompatibility, with an additional function in enriching beneficial *Lactobacillus* in the intestines, supporting its potential application in poultry nutrition to improve iron status and physiological health.

## Figures and Tables

**Table 1:** Composition and nutrient levels of the basal diets for broilers.

Items	1–21 d	22–42 d
Ingredients (%)		
Soybean meal	30.44	31.40
Corn	60.67	54.69
Corn gluten meal	2.00	1.96
Fish meal	2.00	1.88
Soybean oil	3.38	4.68
Dicalcium phosphate	1.50	1.50
Limestone	1.30	1.30
Salt	0.30	0.30
L-Lysine (98%)	0.01	0.01
DL-Methionine (98%)	0.14	0.14
L-Threonine (98%)	0.01	0.01
Premix ^1^	0.50	0.50
Total	100.00	100.00
Measured and calculated nutrient levels ^2^		
Metabolizable energy (kcal/kg)	2950	3153
Crude protein (%)	22.99	19.00
Calcium (%)	0.95	0.90
Total phosphorus (%)	0.37	0.35
SID Lysine (%)	1.40	1.00
SID methionine (%)	0.74	0.38
SID methionine + cystine (%)	1.06	0.70
SID threonine (%)	0.92	0.74
Measured iron content (mg/kg)	35.2	38.1

Notes: ^1^ Premix provided the following per kilogram of diet: Vitamin A, 12,500 IU; Vitamin D_3_, 2500 IU; Vitamin E, 30 mg; Vitamin K_3_, 2.0 mg; Vitamin B_1_, 2.0 mg; Vitamin B_2_, 6.0 mg; Vitamin B_6_, 3.5 mg; Vitamin B_12_, 0.02 mg; Pantothenic acid, 12 mg; Niacin, 35 mg; Folic acid, 1.2 mg; Biotin, 0.20 mg; Choline, 500 mg; Mn (MnSO_4_·H_2_O), 60 mg; Zn (ZnSO_4_·H_2_O), 40 mg; Cu (CuSO_4_·5H_2_O), 8 mg; I (KI), 0.35 mg; and Se (Na_2_SeO_3_), 0.15 mg. Iron was excluded from the premix and supplemented separately as FeSO_4_·H_2_O or nFeS depending on the treatment. ^2^ Nutrient levels were calculated based on NRC (1994) recommendations, except for crude protein, calcium, total phosphorus and iron, which were measured by chemical analysis. SID: Standardized Ileal Digestible.

**Table 2:** Effects of dietary nFeS on the hematological parameters, serum lipid profiles, and antioxidant capacity of broilers.

Items	CON	ID	nFeS			SEM	*p*-Value
			L-FeS	M-FeS	H-FeS		
Day 21							
Hb (g/L)	107.5 ^b^	83.2 ^d^	88.7 ^d^	101.4 ^c^	114.8 ^a^	3.12	<0.001
PCV (%)	32.8 ^b^	25.8 ^d^	26.9 ^d^	30.8 ^c^	35.2 ^a^	1.15	<0.001
TC (mmol/L)	3.02	3.08	3.05	2.97	2.99	0.22	0.798
TG (mmol/L)	0.44	0.48	0.45	0.42	0.45	0.06	0.731
HDL-C (mmol/L)	2.04	1.98	2.00	2.11	2.08	0.08	0.541
LDL-C (mmol/L)	0.70	0.75	0.73	0.68	0.69	0.04	0.437
Day 42							
Hb (g/L)	121.7	115.0	117.1	123.2	126.3	3.58	0.156
PCV (%)	36.5	34.2	35.0	36.9	38.2	1.1	0.171
TC (mmol/L)	2.64	2.77	2.70	2.59	2.63	0.11	0.239
TG (mmol/L)	1.11	1.20	1.15	1.07	1.12	0.08	0.298
HDL-C (mmol/L)	1.82	1.75	1.79	1.86	1.83	0.09	0.403
LDL-C (mmol/L)	0.63	0.67	0.65	0.61	0.63	0.05	0.284
SOD (U/mL)	126.2 ^bc^	118.5 ^c^	123.8 ^bc^	131.1 ^b^	143.5 ^a^	3.25	0.003
GSH-Px (U/mL)	414.6 ^b^	392.1 ^c^	404.4 ^bc^	428.5 ^b^	455.2 ^a^	8.8	0.008
MDA (nmol/mL)	3.14 ^b^	3.58 ^a^	3.34 ^ab^	2.85 ^c^	2.42 ^d^	0.14	0.004

Notes: ^a–d^—the means within a row with different superscripts differ significantly (*p* < 0.05) (*n* = 6). Hb, hemoglobin; PCV, packed cell volume; TC, total cholesterol; TG, triglycerides; HDL-C, high-density lipoprotein cholesterol; LDL-C, low-density lipoprotein cholesterol; SOD, superoxide dismutase; GSH-Px, glutathione peroxidase; MDA, malondialdehyde.

**Table 3:** Effects of nFeS supplementation on serum, digesta, and tissue iron content in AA broilers.

Items	CON	ID	nFeS			SEM	*p*-Value
			L-FeS	M-FeS	H-FeS		
Tissue iron (mg/kg)							
Liver (42 d)	320.5 ^c^	245.8 ^d^	335.8 ^c^	389.6 ^b^	452.7 ^a^	24.9	0.002
Spleen (42 d)	212.3 ^cd^	156.4 ^d^	228.4 ^c^	268.9 ^b^	318.6 ^a^	18.6	0.005
Duodenum (42 d)	82.6 ^b^	61.3 ^c^	80.7 ^bc^	92.6 ^b^	108.4 ^a^	6.9	0.011
Kidney (42 d)	248.5	221.4	230.6	255.2	261.4	18.1	0.128
Intestinal digesta iron (mg/kg DM)							
Duodenal digesta (42 d)	82.8	55.6	61.8	68.9	75.2	7.8	0.135
Jejunal digesta (42 d)	71.8 ^a^	14.2 ^c^	25.8 ^bc^	39.6 ^b^	43.9 ^b^	4.8	<0.001
Cecal digesta (42 d)	26.5 ^a^	5.3 ^d^	9.2 ^cd^	13.0 ^bc^	15.9 ^b^	1.9	<0.001
Serum iron (μmol/L)							
21 d	25.1 ^ab^	19.2 ^c^	21.6 ^bc^	25.4 ^ab^	28.2 ^a^	1.12	0.008
42 d	26.4 ^b^	20.1 ^c^	23.1 ^bc^	27.8 ^b^	32.0 ^a^	1.25	0.004

Notes: ^a–d^—the means within a row with different superscripts differ significantly (*p* < 0.05) (*n* = 6). DM, dry matter; 21 d, day 21 of the trial; 42 d, day 42 of the trial.

**Table 4:** Effects of dietary nFeS supplementation on slaughter performance and meat quality of broilers.

Items	CON	ID	nFeS			SEM	*p*-Value
			L-FeS	M-FeS	H-FeS		
Slaughter performance (%)							
Breast muscle yield	5.48	5.43	5.46	5.53	5.50	0.11	0.662
Thigh muscle yield	6.74	6.63	6.69	6.86	6.83	0.15	0.241
Breast meat color (24 h)							
Lightness (L*)	50.8 ^b^	49.2 ^c^	50.1 ^bc^	52.1 ^a^	51.8 ^a^	0.46	0.018
Redness (a*)	4.4 ^ab^	3.1 ^b^	3.8 ^b^	5.3 ^a^	4.9 ^a^	0.22	0.021
Yellowness (b*)	11.6	12.2	11.9	11.3	11.5	0.45	0.346
Muscle pH							
Breast pH (45 min)	5.83 ^ab^	5.71 ^b^	5.74 ^b^	5.92 ^a^	5.89 ^a^	0.06	0.016
Breast pH (24 h)	5.82	5.77	5.79	5.85	5.83	0.06	0.418
Thigh pH (45 min)	6.18	6.14	6.15	6.22	6.20	0.07	0.552
Thigh pH (24 h)	6.26	6.22	6.24	6.30	6.28	0.08	0.611
Physical properties							
Drip loss (%)	4.32 ^b^	4.98 ^a^	4.72 ^ab^	4.08 ^b^	4.11 ^b^	0.32	0.031
Shear force (N)	376	392	386	370	373	13.1	0.462

Notes: ^a–c^—the means within a row with different superscripts differ significantly (*p* < 0.05) (*n* = 6). L*, lightness; a*, redness; b*, yellowness; pH_45min_, muscle pH at 45 min postmortem; pH_24h_, muscle pH at 24 h postmortem.

**Table 5:** Tolerance evaluation of high-dose dietary nFeS supplementation on the growth performance.

Items	SS	S200	S400	SEM	*p*-Value
1–21 d					
ADG (g/d)	29.12	29.05	28.41	0.90	0.632
ADFI (g/d)	41.24	41.22	40.79	1.15	0.801
FCR	1.42	1.41	1.45	0.05	0.596
22–42 d					
ADG (g/d)	67.08	66.92	65.94	1.35	0.548
ADFI (g/d)	105.23	105.71	104.92	3.10	0.772
FCR	1.56	1.57	1.54	0.07	0.812
1–42 d					
ADG (g/d)	45.18	45.12	44.31	0.85	0.351
ADFI (g/d)	73.22	73.48	72.95	2.05	0.756
FCR	1.59	1.55	1.57	0.05	0.523

Notes: ADG, average daily gain; ADFI, average daily feed intake; FCR, feed conversion ratio; SEM, standard error of the mean.

**Table 6:** Tolerance evaluation of high-dose dietary nFeS supplementation on the hematological parameters.

Items	SS	S200	S400	SEM	*p*-Value
Day 21					
Hb (g/L)	109.4	111.2	107.8	3.50	0.712
PCV (%)	33.6	32.9	34.1	1.35	0.650
MCV (fL)	92.2	91.0	93.1	2.10	0.755
MCH (pg)	31.7	32.2	31.1	1.05	0.680
WBC (×10^9^/L)	18.24	19.62	18.73	1.15	0.284
PLT (×10^9^/L)	246	258	249	14.50	0.520
Day 42					
Hb (g/L)	118.7	116.9	119.5	3.85	0.775
PCV (%)	36.3	35.4	36.8	1.48	0.692
MCV (fL)	94.6	95.2	93.8	2.22	0.725
MCH (pg)	32.5	31.8	32.9	1.10	0.705
WBC (×10^9^/L)	19.45	21.02	20.16	1.30	0.185
PLT (×10^9^/L)	267	281	272	15.80	0.465

Notes: Hb, hemoglobin; PCV, packed cell volume; MCV, mean corpuscular volume; MCH, mean corpuscular hemoglobin; WBC, white blood cell count; PLT, platelet count; SEM, standard error of the mean.

**Table 7:** Tolerance evaluation of high-dose dietary nFeS supplementation on the hepatic and renal functional makers, and antioxidant status.

Items	SS	S200	S400	SEM	*p*-Value
Hepatic and Renal Function					
ALT (U/L)	18.42	20.13	19.21	1.75	0.248
AST (U/L)	196.35	205.42	199.76	8.50	0.276
T-BIL (μmol/L)	3.21	3.48	3.31	0.25	0.745
TP (g/L)	32.84	33.42	32.67	1.30	0.850
ALB (g/L)	14.27	13.98	14.36	0.58	0.795
BUN (mmol/L)	3.87	4.06	3.92	0.30	0.715
CREA (μmol/L)	35.62	36.88	35.97	2.10	0.615
Antioxidant Status					
SOD (U/mL)	145.2	148.7	144.3	5.80	0.415
GSH-Px (U/mL)	312.5	326.4	317.6	11.50	0.370
CAT (U/mL)	18.42	19.26	18.71	0.88	0.295
T-AOC (U/mL)	7.86	8.05	7.92	0.32	0.540
MDA (nmol/mL)	4.12	4.34	4.18	0.30	0.185

Notes: ALT, alanine aminotransferase; AST, aspartate aminotransferase; T-BIL, total bilirubin; TP, total protein; ALB, albumin; BUN, blood urea nitrogen; CREA, creatinine; SOD, superoxide dismutase; GSH-Px, glutathione peroxidase; CAT, catalase; T-AOC, total antioxidant capacity; MDA, malondialdehyde; SEM, standard error of the mean.

**Table 8:** Tolerance evaluation of high-dose dietary nFeS supplementation relative to the organ weights.

Items	SS	S200	S400	SEM	*p*-Value
Liver (g/kg BW)	24.18	24.91	24.36	0.75	0.645
Spleen (g/kg BW)	1.62	1.71	1.65	0.08	0.610
Kidney (g/kg BW)	6.12	6.03	6.24	0.18	0.782

Notes: BW, body weight; SEM, standard error of the mean.

## Data Availability

The data presented in this study are available upon request from the corresponding author. The data are not publicly available, for privacy-related reasons.

## Supplementary Materials

The following supporting information can be downloaded at: https://www.mdpi.com/article/10.3390/ani16172660/s1, Table S1: nFeS effects on growth performance of AA broilers; Table S2: nFeS effects on immune organ indices of AA broilers.
