# Supplementary material for: Effects of Nano-Iron Sulfide on Growth Performance, Antioxidant Status, Meat Quality, and Intestinal Microbiota in Broilers, and Tolerance Evaluation
Source: Animals (Basel). 2026 Aug 25;16(17):2660. doi: 10.3390/ani16172660 (PMC13564325; doi:10.3390/ani16172660)
Supplement: Supplementary file 1 [file animals-16-02660-s001.zip › animals-4479918-supplementary.pdf]

Table S1. nFeS effects on growth performance of AA broilers.

Items	CON	ID	nFeS			SEM	*P*-value
			L-FeS	M-FeS	H-FeS		
**Starter (1–21 d)**							
ADG (g/d)	28.92[b]	22.08[c]	23.45[c]	30.18[ab]	32.42[a]	1.25	<0.001
ADFI (g/d)	40.50	39.16	39.88	41.85	43.56	1.42	0.185
FCR	1.44[b]	1.77[a]	1.70[a]	1.40[b]	1.35[b]	0.05	<0.001
**Grower (22–42 d)**							
ADG (g/d)	66.20	64.81	65.12	67.18	68.96	1.52	0.452
ADFI (g/d)	104.49	112.08	108.55	106.88	111.94	3.54	0.58
FCR	1.58	1.68	1.65	1.55	1.51	0.07	0.215
**Overall (1–42 d)**							
ADG (g/d)	44.89	42.67	43.15	45.12	46.88	0.95	0.072
ADFI (g/d)	72.49	75.62	74.21	73.41	76.78	2.18	0.642
FCR	1.62[b]	1.77[a]	1.72[a]	1.61[b]	1.56[b]	0.05	0.045

Notes:

[a–c] Means within a row with different superscripts differ significantly ($P < 0.05$) (n = 6).

ADG, average daily gain; ADFI, average daily feed intake; FCR, feed conversion ratio.

Table S2. nFeS effects on immune organ indices of AA broilers.

Items	CON	ID	nFeS			SEM	*P*-value
			L-FeS	M-FeS	H-FeS		
**Spleen (g/kg)**							
21 d	0.178	0.162	0.169	0.182	0.176	0.015	0.315
42 d	0.180	0.165	0.172	0.188	0.185	0.014	0.158
**Thymus (g/kg)**							
21 d	0.325	0.298	0.312	0.308	0.322	0.022	0.246
42 d	0.332	0.302	0.320	0.328	0.326	0.025	0.182
**Bursa of Fabricius (g/kg)**							
21 d	0.485	0.452	0.458	0.470	0.488	0.035	0.211
42 d	0.374	0.335	0.360	0.382	0.375	0.030	0.082

Notes:

SEM, standard error of the mean. Data are presented as means of six replicates per treatment (n = 6).
